# Unveiling the gut microbiota composition and functionality associated with constipation through metagenomic analyses

**DOI:** 10.1038/s41598-017-10663-w

**Published:** 2017-08-29

**Authors:** Leonardo Mancabelli, Christian Milani, Gabriele Andrea Lugli, Francesca Turroni, Marta Mangifesta, Alice Viappiani, Andrea Ticinesi, Antonio Nouvenne, Tiziana Meschi, Douwe van Sinderen, Marco Ventura

**Affiliations:** 1 Laboratory of Probiogenomics, Department of Chemistry, Life Sciences, and Environmental Sustainability, University of Parma, Parma, Italy; 2 GenProbio srl, Parma, Italy; 3 Department of Medicine and Surgery, University of Parma, Italy; Dipartimento Medico-Geriatrico-Riabilitativo, Azienda Ospedaliero-Universitaria di Parma, Parma, Italy; 4 APC Microbiome Institute and School of Microbiology, Bioscience Institute, National University of Ireland, Cork, Ireland

## Abstract

Functional constipation (FC) is a gastrointestinal disorder with a high prevalence among the general population. The precise causes of FC are still unknown and are most likely multifactorial. Growing evidence indicates that alterations of gut microbiota composition contribute to constipation symptoms. Nevertheless, many discrepancies exist in literature and no clear link between FC and gut microbiota composition has as yet been identified. In this study, we performed 16 S rRNA-based microbial profiling analysis of 147 stool samples from 68 FC individuals and compared their microbial profiles with those of 79 healthy subjects (HS). Notably, the gut microbiota of FC individuals was shown to be depleted of members belonging to *Bacteroides, Roseburia* and *Coprococcus 3*. Furthermore, the metabolic capabilities of the gut microbiomes of five FC and five HS individuals were evaluated through shotgun metagenomics using a MiSeq platform, indicating that HS are enriched in pathways involved in carbohydrate, fatty acid and lipid metabolism as compared to FC. In contrast, the microbiomes corresponding to FC were shown to exhibit high abundance of genes involved in hydrogen production, methanogenesis and glycerol degradation. The identified differences in bacterial composition and metabolic capabilities may play an important role in development of FC symptoms.

## Introduction

The human gastrointestinal tract is colonized by complex communities of microorganisms, i.e. the gut microbiota, that are involved in several physiological functions of the host. These encompass metabolic, nutritional, physiological and immunological processes that are vital to maintain the host’s health status^1, 2^. In this context, alterations in the gut microbiota composition have been linked to certain common human intestinal diseases, such as pseudomembranous colitis (CDI)^3–8^, ulcerative colitis (UC)^9–11^ and Crohn’s disease (CD)^9, 12, 13^. However, changes in the gut microbiota composition are also considered to play a crucial role in the establishment of gut related disorders such as irritable bowel syndrome (IBS)^14–16^. Functional constipation (FC) is a common gastrointestinal disorder with a prevalence between 5% and 20% of the general population^17, 18^, provoking a significant impact on quality of life^19^. In fact, it can result in discomforts such as abdominal distension, abdominal pain, headache, dizziness and loss of appetite^20^. Despite its high prevalence, only a small number of studies have investigated its possible correlation with particular gut microbiota alterations. Additionally, most of these studies relied on culture-based methods that are unable to assess the unculturable portion of the gut microbiota^21, 22^. Moreover, the functional implications of these alterations and their impact on host physiology have never been assessed. Recently, two metagenomic studies compared the microbial population of stools collected from constipated and healthy individuals, highlighting an altered fecal microbiome associated with constipation^23, 24^. However, these studies were limited by the small sample size and heterogeneity of participants (including women or obese children). Furthermore, it is worth mentioning that the observed differences in microbiota composition among healthy and constipated patients suffer from a number of discrepancies^20^.

Here, in order to identify a statistically significant and comprehensive correlation between microbiota and constipation, we performed 16 S rRNA-based profiling analysis of 147 stool samples collected from 68 functional constipated (FC) and 79 healthy subjects (HS). Furthermore, in order to better understand the role of the microbiome and its metabolic impact on the host, the gut microbiome of a random subsampling of 10 samples, five FC and five HS samples, was reconstructed and analyzed in detail by shotgun metagenomic analyses.

## Results and Discussion

### Patient enrollment and collection of fecal samples

In this study, we collected and analyzed 147 human stool samples from Italian subjects. More specifically, we obtained 68 samples from individuals affected by functional constipation (FC), while 44 samples were collected from healthy subjects (HS). The HS dataset was supplemented with data from 35 samples that we published previously^8, 25^. Notably, these samples had been collected and processed using the same protocols as followed for the 112 samples sequenced in the current study (see below for details). Moreover, analysis of variance of beta-diversity was performed between each pool of samples processed in different sequencing runs, and the obtained data showed absence of any batch effects (Fig. S1a). In order to identify microbial biomarkers of functional constipation across all age ranges, we selected individuals with an age ranging from 4 to 94 (average age: 42 ± 22 years) (Table S1). Remarkably, beta-diversity and PERMANOVA analyses displayed absence of age-related clustering of the samples (Fig. S1b). Moreover, the enrolled individuals were not taking prebiotics and/or probiotics, not undergoing antibiotic treatment or any other medical therapy (including those specific for functional constipation such laxatives for one week prior sampling) and not suffering from acute or severe intestinal diseases such as ulcerative colitis (UC), Crohn’s disease, acute inflammatory bowel disease (IBD), intestinal cancer and enteritis. Notably, functionally constipated individuals also reporting symptoms typical of IBS-C, such as abdominal pain, were excluded from this study. The selected individuals affected by functional constipation fulfill the ROME-III criteria and manifested infrequent bowel movements that are defined as three or less defecations per week^26^. Notably, statistical assessment of diet homogeneity of FC and HS groups revealed absence of statistically significant differences (Table S2). In order to avoid discrepancies in the *in silico* data, all newly sequenced as well as previously published datasets included in this study were subjected to bioinformatic analysis using the same pipeline based on a custom script for the Qiime software suite and the same 16 S rRNA database (see Methods for details).

### Intra- and Inter-individual variability among healthy and functionally constipated subjects

Stool samples from the 147 individuals enrolled in this study were obtained in order to assess the microbiota composition based on 16 S rRNA-based sequencing analysis, as described previously^27^. MiSeq-mediated sequencing of the samples produced a total of 18,673,728 reads with an average of 127,032 ± 69,090 reads per sample (Table S2). Quality and chimera filtering produced a total of 10,164,847 filtered reads with an average of 69,149 filtered reads per sample, and ranging from 185,347 to 10,440 reads (Table S2).

**Figure 1:**
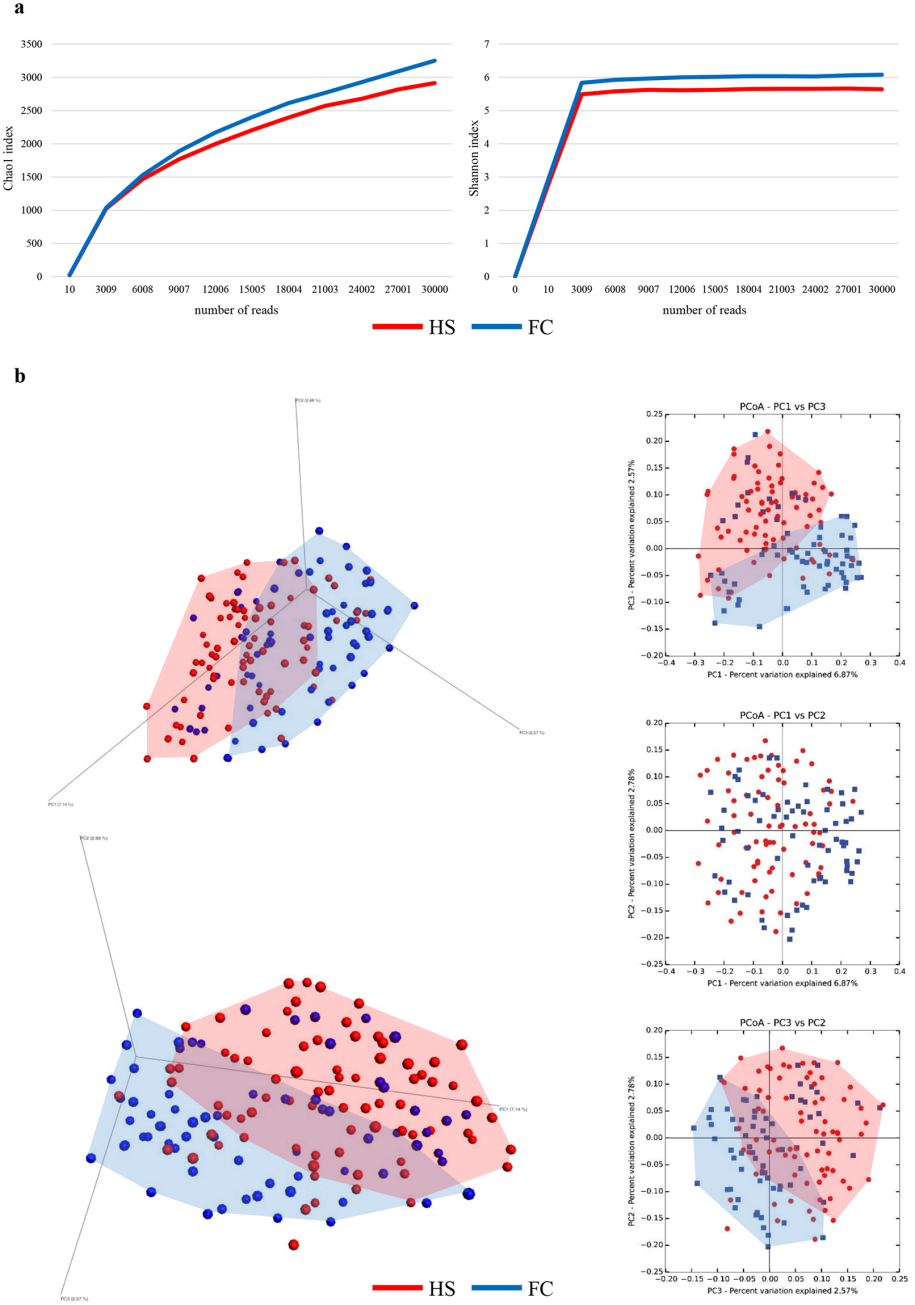
Evaluation of the alpha- and beta-diversity in the 147 analyzed samples. Panel a reports the average rarefaction curves based on the Chao1 and Shannon index at increasing sequencing depth of FC and HS samples. Panel b shows the predicted PCoA through two three-dimensional images and two-dimensional sections. FC and HS datasets and corresponding clusters are colored in blue and red, respectively.

Evaluation of rarefaction curves obtained through the Shannon and Chao1 biodiversity indeces calculated for 10 sub-samplings of sequenced read pools showed that both curves tend to reach a plateau. Therefore, in all cases the retrieved sequencing data is considered adequate to cover the vast majority of biodiversity contained within the samples (Fig. 1). Interestingly, average rarefaction curves revealed a difference between FC and HS samples in that, on average, the former samples were shown to exhibit a higher level of gut microbiota complexity compared to the latter samples. Statistical analysis, calculated for the highest sub-sampling point reached by all samples, i.e. 30,000 reads, showed that the two curves significantly differ based on a one-way analysis of variance (ANOVA) (p-value < 0.05). The observed dissimilarity of the alpha-diversity between FC and HS is in accordance with a previous study^24^, being indicative of differences in bacterial composition and corresponding metabolic potential (see below). In order to evaluate the inter-individual differences between FC and HS samples in more detail, we assessed the beta-diversity^28^ by means of unweighted UniFrac^29^ and represented the results through a 3-Dimensional Principal Coordinate Analysis (PCoA). The PCoA plot shows that the majority of the samples are grouped as two different clusters that correspond to FC or HS individuals, thus highlighting an intriguing difference in microbiota composition between individuals that suffer from functional constipation and healthy individuals (Fig. 1). Notably, the obtained results were statistically supported by PERMANOVA analyses (p-value of <0.001).

### Taxonomic profiling of the gut microbiota of functionally constipated and healthy individuals

Inspection of the predicted taxonomic profiles showed that both FC and HS samples possess a preponderant presence of the phyla Bacteroidetes (50.44% ± 18.29% and 58.37% ± 16.59%, respectively; p-value < 0.05) and Firmicutes (44.19% ± 17.02% and 36.65% ± 15.65%, respectively; p-value < 0.05), although with a significantly different average relative abundance (Fig. S2). When analyzed at genus level, the FC group displayed high levels of *Bacteroides* (34.25% ± 18.56%), *Faecalibacterium* (6.85% ± 6.19%), *Alistipes* (6.48% ± 9.69%), *Lachnospira* (4.44% ± 6.13%) and Unclassified member of Lachnospiraceae family (3.92% ± 2.40%). Similarly, the most represented taxa detected in HS samples were *Bacteroides* (45.23% ± 17.90%), *Alistipes* (5.34% ± 5.60%) and Unclassified member of Lachnospiraceae family (4.66% ± 4.38%).

Comparative analysis of the 331 bacterial taxa predicted by genus-level analysis revealed that 23 genera appeared to be present only in HS samples, while 17 were uniquely present in FC subjects (Table. S3). Analysis of the proportion of these unique genera found in each group, i.e. the prevalence, showed that these taxa are present in <20% of FC or HS profiles, thus indicating the absence of specific microbial biomarkers whose presence or absence is associated with constipation while pointing at a probable role played by the overall gut microbiota at functional level.

### Difference in gut microbiota composition

**Figure 2:**
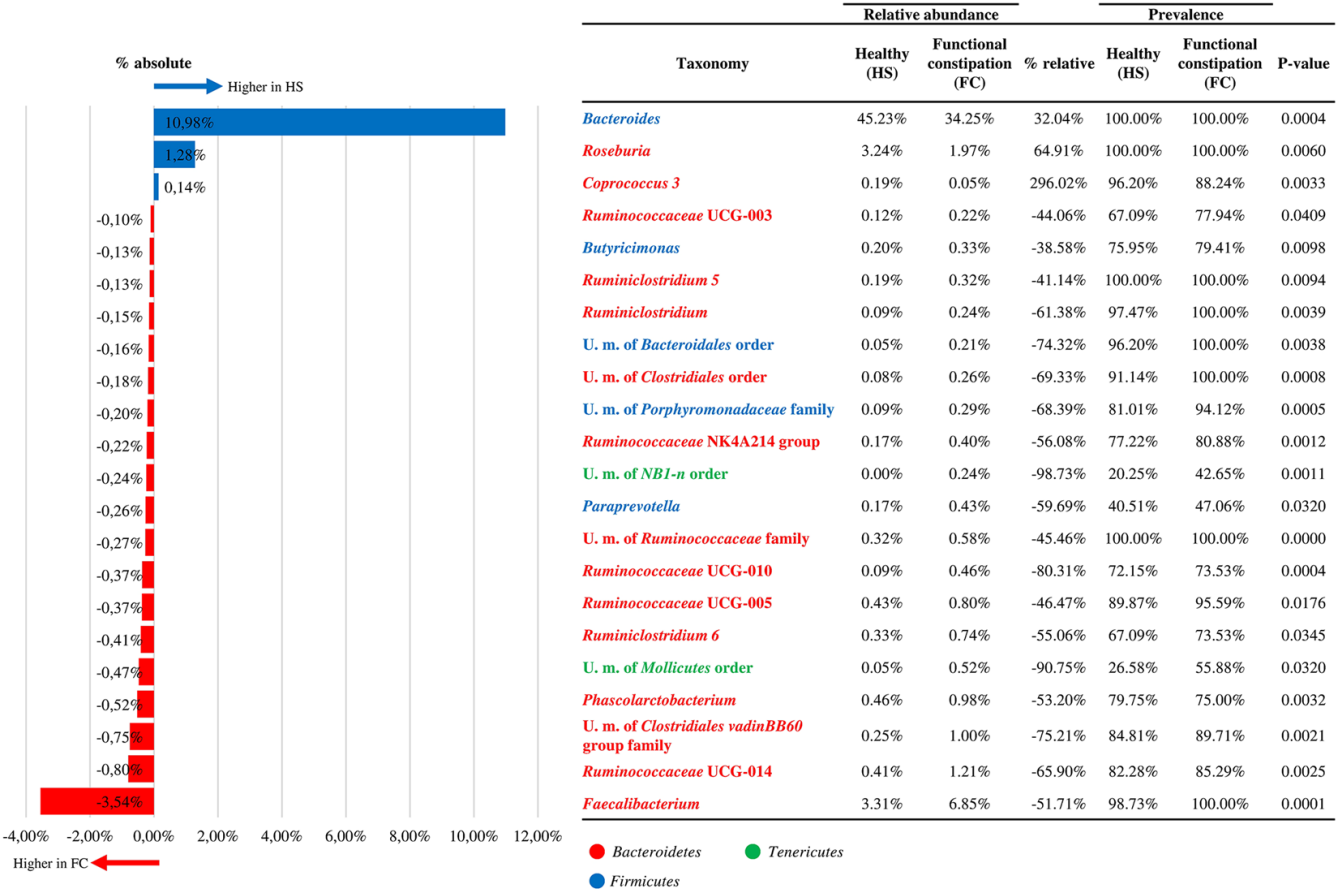
Exploration of the diversity in HS and FC groups. The bar plot reports only genera with an absolute percentage difference between HS and FC averages >0.1% and a p-value < 0.05, evaluated by means of ANOVA statistical analysis. The table indicates the bacterial genera, the relative abundance and the prevalence of each group, the relative percentage difference and the p-value.

In order to evaluate possible differences in bacterial composition, ANOVA statistical analysis was employed to compare the average relative abundance in FC and HS groups of genera with an absolute percentage difference >0.1% (Fig. 2). Interestingly, the comparison between HS and FC datasets showed that profiles obtained from HS individuals are characterized by a statistically significant over-representation of *Bacteroides* (% absolute 1.28%, p-value < 0.01), *Roseburia* (% absolute 1.28%, p-value < 0.01) and *Coprococcus 3* (% absolute 0.14%, p-value < 0.01) and a statistically significant under-representation of genera belonging to the Ruminococcaceae family such as *Faecalibacterium* (% absolute −3.54%, p-value < 0.01) (Fig. 2). The depletion of the *Bacteroides* genus in FC samples may be correlated with alterations of the intestinal motility and secretory functions due to changes in the amount of available physiologically active substances in the metabolic environment of the gut^20, 30^. In fact, the higher abundance of butyrate-producing taxa, such as *Coprococcus* and *Roseburia*, observed in HS samples may explain a faster colonic transit due to the motility-stimulating effect exerted by butyrate in the gut^1^. In this context, previous studies have reported that butyrate-producing taxa may stimulate colonic motility by induction of serotonin release or by facilitating cholinergic pathways by means of butyrate production^31, 32^. In contrast, despite being a butyrate-producer, *Faecalibacterium* is significantly more abundant in FC samples. Interestingly, this genus has been reported to contribute to the pathogenesis of constipation via several mechanisms, such as inhibition of mucin secretion and reduction of stool volume^24^.

### Metagenomic characterization of FC and HS microbiomes

**Figure 3:**
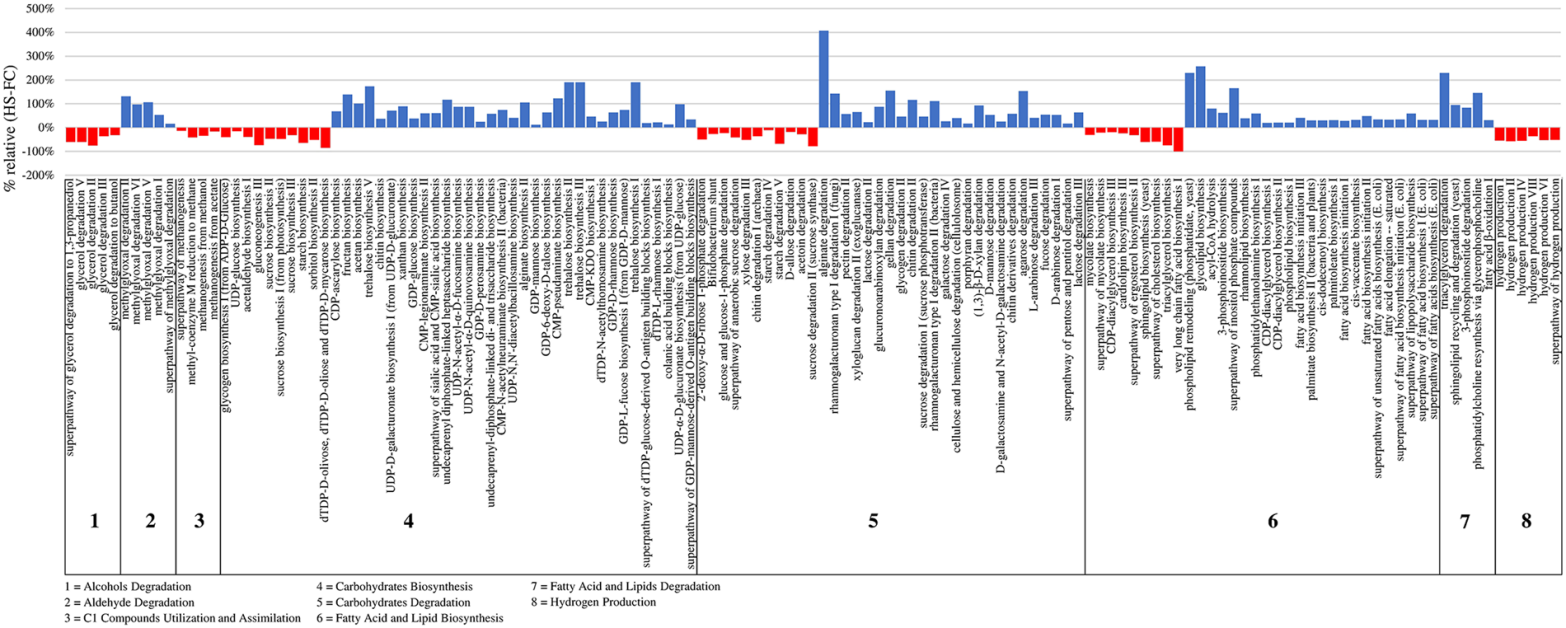
Functional characterization of FC and HS microbiomes. The bar plot shows the relative difference between the average abundance in HS and FC subjects of each pathway displaying ANOVA with p-value < 0.05.

A total of 10 individuals were selected among the two clusters obtained from the PCoA analysis (Fig. 1b) as representatives of the FC and HS groups, and total bacterial DNA extracted from corresponding fecal samples was subjected to Illumina shotgun sequencing. These samples were selected on the basis of their microbial profiles that were shown to be closer to the average for each group. Collected read pools ranged from 8,656,289 to 2,885,092 after quality filtering, with an average number of reads per sample of 6,130,802 (Table S4). These data were then utilized for the reconstruction of metabolic pathway profiles in the analyzed microbiomes by means of a custom script based on the MetaCyc database^33^. A comparison between the averages of functionally constipated and the averages of healthy samples showed a significant difference in 629 pathways (p-value < 0.05). Of the latter, 327 and 302 pathways were more abundant in FC and HS samples, respectively (Table S5). Interestingly, the HS samples exhibited a higher abundance of genes (p-value < 0.05) involved in carbohydrate (increase of 21.15%) and fatty acid metabolism (increase of 25.93%) as compared to FC individuals (Fig. 3b). These pathways are implicated in production of short-chain fatty acids (SCFAs) and may play a role in stimulating ileal propulsive contractions through an enteric cholinergic reflex, thereby counteracting functional constipation^20, 34–36^. Moreover, FC samples were shown to contain a higher abundance of genes involved in methanogenic pathways (increase of 24.96%, p-value < 0.01) and a predicted higher capability to produce hydrogen (increase of 113.69%, p-value 0.05) as compared to HS (Table S5, Fig. 3b). Thus, our data are consistent with previous observations and suggest that the (abundant) presence of methanogenic and H_2_-consuming populations influence colonic motility and visceral sensitivity, and generate chronic constipation along with several correlated symptoms, such as flatulence and abdominal distension^37, 38^. Interestingly, datasets obtained from functionally constipated individuals showed lower abundance of genes involved in methylglyoxal degradation as compared to healthy samples (decrease of −25.18%, p-value < 0.05) (Fig. 3b). Methylglyoxal is produced by intestinal bacteria and it is reported be a potential toxic metabolite that can be involved in many gut diseases, including functional constipation^39^. Furthermore, comparison between HS and FC samples highlights a significant difference in pathways implicated in glycerol degradation (decrease of −58.93% in HS, p-value < 0.05) (Fig. 3b). Glycerol is known to cause an osmotic effect in the rectum and for this reason is used to treat constipation^40^. Therefore, the increased ability to degrade glycerol by FC samples may induce and promote the symptoms of constipation. Nevertheless, despite the fact that the use of laxatives in the week before sampling was considered as a exclusion criterion, this observation may be linked to previous and prolonged use of PEG or glycerol suppositories.

**Figure 4:**
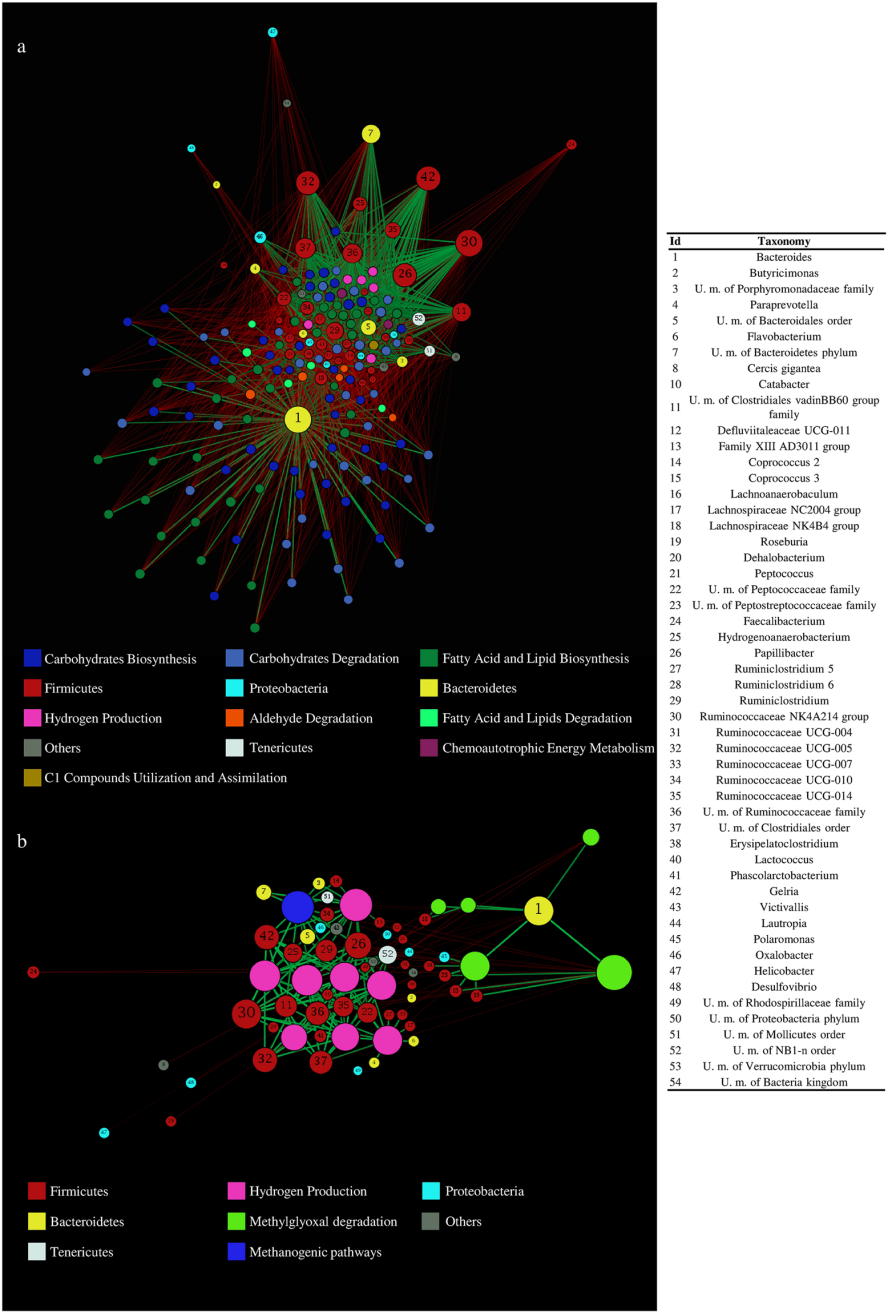
Co-variance network of bacterial genera and metabolic pathways with statistically significant difference in abundance between FC and HS subjects. Panel a shows a force-driven network based on the predicted co-variances with p-values < 0.05 between the genera and metabolic pathways identified as statistically altered in FC as compared to HS. Panel b reports a force-driven network based on the predicted co-variances with p-values < 0.05 between genera and metabolic pathways putatively involved in FC development. Co-variances with p-values < 0.05 are used to define the attractive or repulsive force of the edges. The node size is proportional to the number of co-variances. Node color indicates phylum or pathway category as reported in the image. The taxonomy, i.e. bacterial genera, of the nodes is indicated with number, as listed in the table.

In addition, we evaluated co-variance between the bacterial genera and metabolic pathways displaying difference in abundance (ANOVA p-value < 0.05) in FC as compared to HS subjects. Force-driven network representation of these data revealed that *Bacteroides* (124 co-variances, p-value < 0.05), genera belonging Ruminococcaceae family (a total of 270 co-variances, p-value < 0.05) and the methanogenic genus *Gelria* (108 interactions, p-value < 0.05) exert a key role in modulating the metabolic functionalities of the gut microbiome that are altered during FC (Fig. 4a).

Moreover, a force-driven network representation based on the above-discussed pathways that may be related to FC development revealed that the Firmicutes phylum positively correlates with pathways involved in hydrogen production and methanogenesis, while *Bacteroides* correlates positively with the methylglyoxal degradation pathways (Fig. 4b). These results reflect the putative beneficial role played by *Bacteroides* to counter FC as also suggested by the higher abundance of this genus in HS with respect to FC samples (see above). Notably, an in depth analysis of 16 S rRNA OTUs of HS samples revealed that 17.33% of the reads belonging to the *Bacteroides* genus correspond to unknown species, followed by *Bacteroides vulgatus* (13.83%), *Bacteroides uniformis* (4.21%) and *Bacteroides fragilis* (1.74%), thus indicating that further genomic analyses are still needed to shed light on the biological role of this genus in protecting against or in preventing FC.

## Conclusions

Functional constipation is a widespread gastrointestinal disorder responsible for difficult or infrequent bowel movements defined as three or less defecations per week^26^. Despite the high worldwide prevalence of FC, a clear anatomical or physiological cause for this disorder has yet to be identified, thus pointing at a possible role exerted by the gut microbiota. Here, we confirm preliminary findings regarding gut microbiota compositional shifts in individuals affected by FC as compared to healthy controls, which are concurrent with a statistically significant increase of the gut microbiota biodiversity. Moreover, statistical analysis revealed alterations in relative abundance of specific taxa, such as *Bacteroides* and *Feacalibacterium*. Disregarding the fact that the cause of such taxonomic changes can’t be linked to a specific physiological cause and may simply reflect altered transit time or diet, these taxa will be pivotal for diagnostic and prophylactic purposes as statistically-supported microbial biomarkers of constipation. Intriguingly, identification of taxa typically associated to a healthy gut status, e.g. the anti-inflammatory genus *Feacalibacterium*, as a biomarker of functional constipation, highlights that increased relative abundance of certain taxa in the presence of a gut disorder may not reflect a taxa-specific role in pathogenesis but may be linked to a global alteration of gut microbiota’s homeostasis. Notably, the taxonomic profiles retrieved from faecal samples include both the autochthonous as well as the allochthonous microbiota, and may thus not be fully representative of the resident gut microbial population. In contrast, the use of biopsies from mucosal samples would be ideal to provide information on the indigenous microbiota^41^. Nevertheless, collection of fecal samples is less invasive and does not require specific clinical procedures. Thus, for a rapid screening aimed at the identification of biomarkers associated with specific disorders, e.g. functional constipation, the use of stool samples does not suffer from the aforementioned problems associated with collection of biopsies.

While exploration of the functional role of the gut microbiota in fecal transit time^42^ has been attempted in the past through analysis of urine metabolites, the whole gut microbiome metabolic potential and its impact on host physiology and development of functional constipation has yet to be elucidated. To overcome this gap, we profiled metabolic pathways of microbiomes corresponding to functionally constipated and healthy individuals. The here reconstructed gut microbiome of individuals affected by functional constipation revealed for the first time that the FC microbiome is characterized by a high abundance of genes involved in hydrogen production, methanogenesis and glycerol degradation. In contrast, the microbiomes of HS samples showed an increase of pathways implicated in carbohydrate and fatty acid metabolism, and in methylglyoxal degradation. Alteration of these metabolic pathways appears to impact on functional constipation and related symptoms, thus highlighting the key functional role exerted by the gut microbiome in maintaining the health status of the host. Nevertheless, due to the limited number of samples that were analysed by shotgun sequencing, additional experiments are needed to validate these observations.

Altogether, taxonomic and functional data reported in this study represent a solid base for future development of both prophylactic screenings and therapies for functional constipation based on alterations of gut microbiota composition through personalized diet or pre- and pro-biotic treatments.

## Methods

### Datasets included in this study

We enrolled 68 volunteers suffering from functional constipation (FC) and 44 healthy subjects (HS) in an outpatient clinic setting. The individuals affected by functional constipation presented infrequent bowel movements and fulfilled the ROME-III criteria. A stool sample, consisting of 6–10 g fresh fecal material, was obtained from each subject and immediately frozen at −80 °C until it was processed for DNA extraction. DNA was extracted from each stool sample using the QIAamp DNA Stool Mini kit (Qiagen Ltd, Strasse, Germany) following the manufacturer’s instructions (Qiagen Ltd). The study protocol was approved by the Ethics Committee of the University of Parma. Informed consent was obtained from all participants. All investigations were carried out following the principles of the Declaration of Helsinki.

### 16 S rRNA gene amplification

Partial 16 S rRNA gene sequences were amplified from extracted DNA using primer pair Probio_Uni and/Probio_Rev, which targets the V3 region of the 16 S rRNA gene sequence^27^. Illumina adapter overhang nucleotide sequences were then added to the partial 16 S rRNA gene-specific amplicons, which in turn were further processed by employing the 16 S Metagenomic Sequencing Library Preparation Protocol (Part no. 15044223 Rev. B—Illumina; see also below). Amplifications were carried out using a Verity Thermocycler (Applied Biosystems). The integrity of the PCR amplicons was analyzed by electrophoresis on a 2200 TapeStation Instrument (Agilent Technologies, USA).

### MiSeq sequencing of 16 S rRNA gene-based amplicons

PCR products obtained following amplification of part of the 16 S rRNA gene sequences were purified by a magnetic purification step involving Agencourt AMPure XP DNA purification beads (Beckman Coulter Genomics GmbH, Bernried, Germany) in order to remove primer dimers. The DNA concentration of the amplified sequence library was estimated through fluorimetric Qubit quantification system (Life Technologies). Amplicons were diluted to 4 nM and 5 μl of each diluted DNA amplicon sample was mixed to prepare the pooled final library. Sequencing was performed using an Illumina MiSeq sequencer with MiSeq Reagent Kit v3 chemicals.

### Analysis of 16 S rRNA microbial profiling datasets

The fastq files were processed using QIIME^28^ as previously described^27^. Paired-end reads were merged, and quality control implementation allowed the retention of sequences with a length between 140 and 400 bp, mean sequence quality score >25 and with truncation of a sequence at the first base if a low quality within a rolling 10-bp window was found. Sequences with mismatched forward and/or reverse primers were omitted. 16 S rRNA operational taxonomic units (OTUs) were defined at ≥ 97% sequence homology using uclust^43^. All reads were classified to the lowest possible taxonomic rank using QIIME^28^ and a reference dataset from the SILVA database v. 123^44^. The microbial richness of the samples (alpha-diversity) was evaluated with the Chao1 and Shannon index through the alpha_rarefaction.py script included in the Qiime software suite using default parameters. Similarities between samples (beta-diversity) were calculated by unweighted uniFrac^45^. Principal coordinate analysis (PCoA) representations of beta-diversity were performed using QIIME^28^.

### Shotgun metagenomics

DNA library preparation was performed using the Nextera XT DNA sample preparation kit (Illumina, San Diego, CA) according to the manufacturer’s instructions. In brief, 1 ng input DNA from each sample was used for library preparation. The isolated DNA underwent fragmentation, adapter ligation and amplification. The ready-to-go libraries were pooled equimolarly, denaturated and diluted to a sequencing concentration of 1.8 pM. Sequencing was performed on NextSeq. 550 instrument (Illumina, San Diego, CA), according to the manufacturer’s instructions, using the 2 × 150 bp High Output sequencing kit, and spike-in of 1% PhiX control library.

### Analysis of metagenomic datasets

The generated paired fastq files were filtered for reads with a quality score of <25, for sequences of human DNA, as well as for reads <80 bp. Bases were also removed from the end of the reads unless the average quality score in a window of 5 bp was >25. Reconstruction of bacterial metabolic pathways was performed using custom scripts based on htseq-count^46^ and the MetaCyc database^33^, respectively.

### Statistical analyses

QIIME and SPSS software (www.ibm.com/software/it/analytics/spss/) were used to complete statistical analysis. All data were presented as means ± SEM. PERMANOVA were performed using 999 permutations to estimate p-values for differences among populations. Furthermore, differential abundance of bacteria taxa and metabolic pathways were tested by one-way analysis of variance (ANOVA).

### Data Deposition

The 16 S rRNA profiling data sequenced in this study were deposited in the Sequence Read Archive (SRA) database under the SRP106879 accession number. Shotgun metagenomics data are accessible through SRA study accession number SRP106935.

## Electronic supplementary material

supplementary information

Supplementary tables
