# Supplementary material for: Unveiling the gut microbiota composition and functionality associated with constipation through metagenomic analyses
Source: Sci Rep. 2017 Aug 29;7:9879. doi: 10.1038/s41598-017-10663-w (PMC5575163; doi:10.1038/s41598-017-10663-w)
Supplement: Supplementary file 1 — supplementary information [file 41598_2017_10663_MOESM1_ESM.pdf]

Unveiling the gut microbiota composition and functionality associated with constipation through metagenomic analyses

Leonardo Mancabelli[1], Christian Milani[1], Gabriele Andrea Lugli[1], Francesca Turroni[1], Marta Mangifesta[1,2], Alice Viappiani[2], Andrea Ticinesi[3], Antonio Nouvenne[3], Tiziana Meschi[3], Douwe van Sinderen[4] and Marco Ventura[1]

Supplementary information

Additional files

Figure S1. Microbial profiling of the 147 samples at phylum level.

Figure S2. Evaluation of batch effects and age incidence on microbiota composition.

Table S1. Fecal samples collected in this study.

Table S2. Evaluation of homogeneity of the samples collected in this study.

Table S3. 16S rRNA microbial profiling data.

Table S4. Taxonomic profiling of the 147 analyzed samples at genus level.

Table S5. Shotgun metagenomic data.

Table S6. List of pathways detected in analysis with a significant difference (p-value < 0.05).

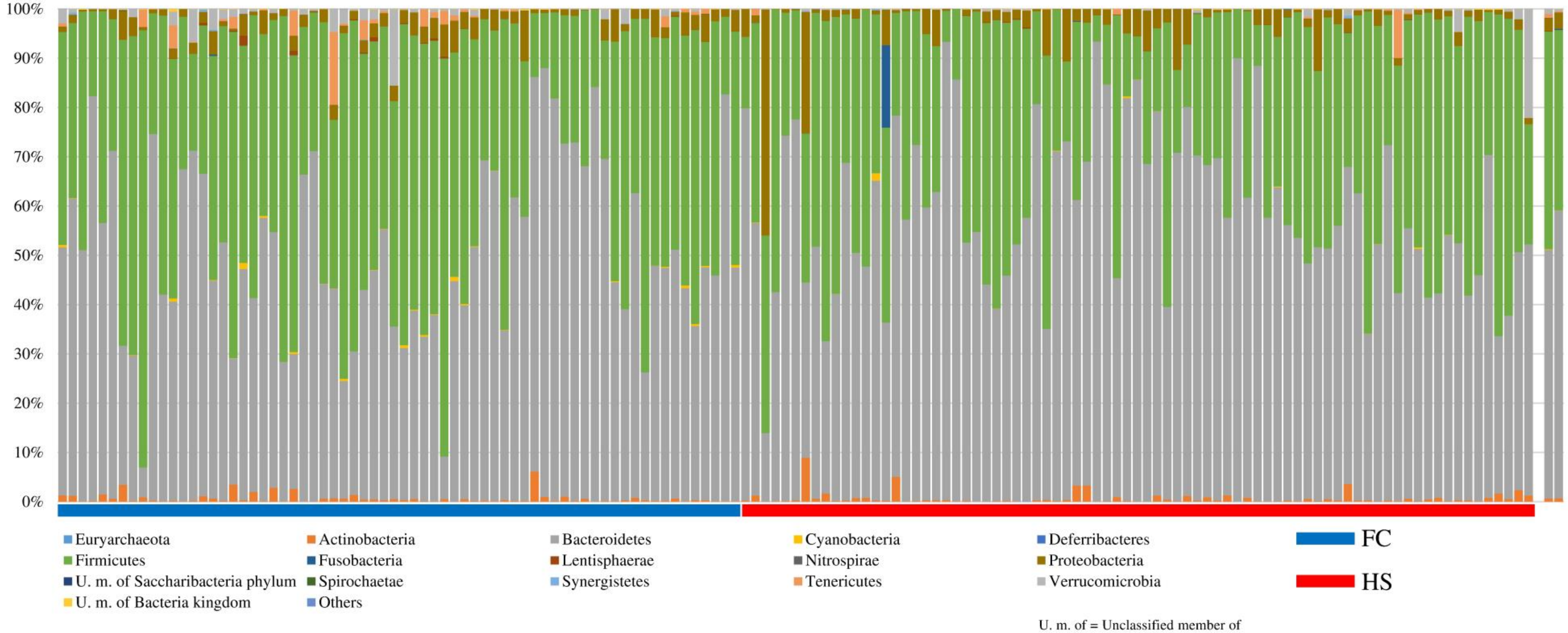
100%
90%
80%
70%
60%
50%
40%
30%
20%
10%
0%
Euryarchaeota
Actinobacteria
Bacteroidetes
Cyanobacteria
Deferribacteres
Firmicutes
Fusobacteria
Lentisphaerae
Nitrospirae
Proteobacteria
U. m. of Saccharibacteria phylum
Spirochaetae
Synergistetes
Tenericutes
Verrucomicrobia
U. m. of Bacteria kingdom
Others
FC
HS
U. m. of = Unclassified member of

Figure S1

a

b

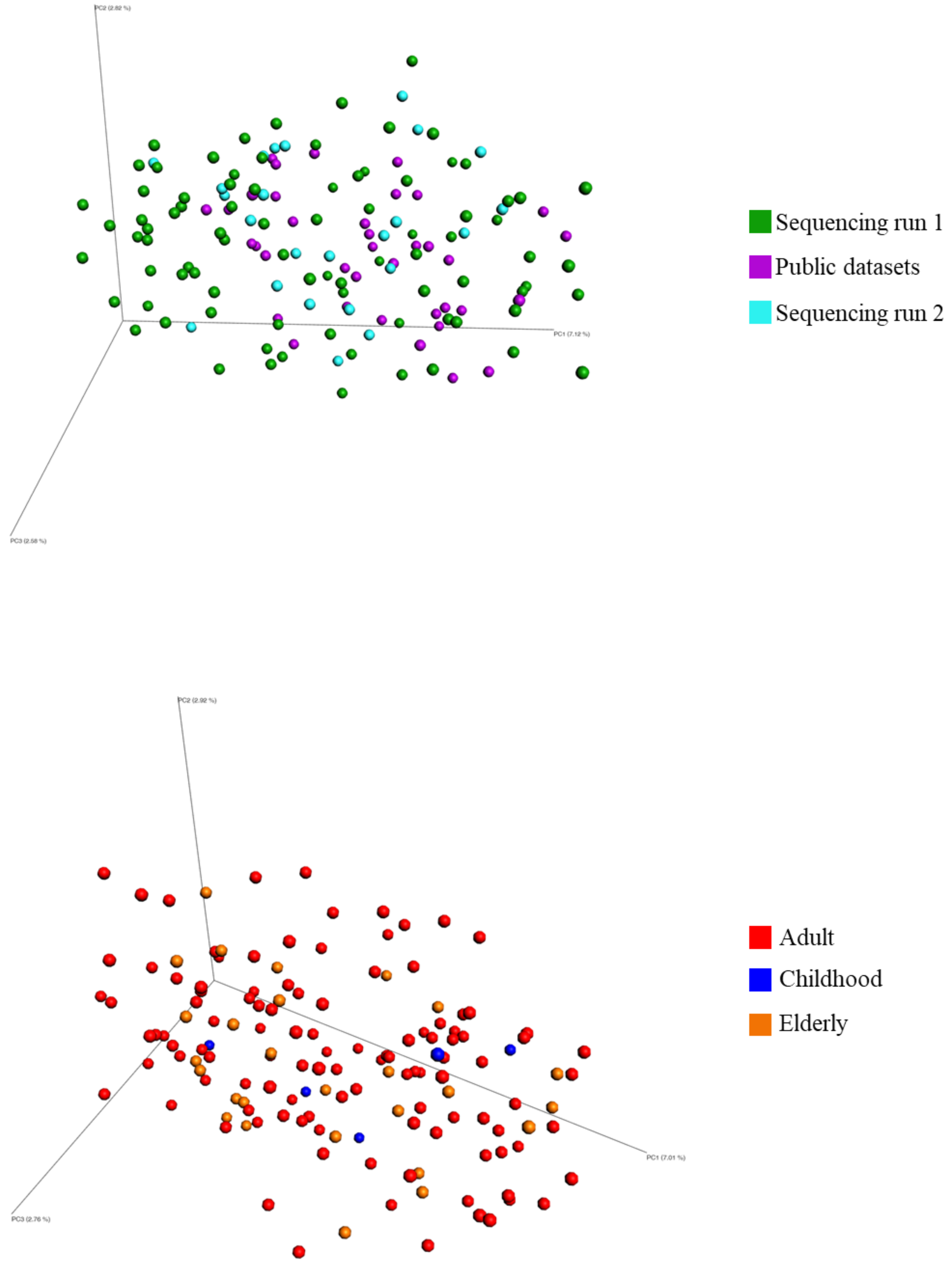

Sequencing run 1
Public datasets
Sequencing run 2
Adult
Childhood
Elderly

Figure S2
